# Leiomyosarcoma of the spermatic cord: A case report and literature review

**DOI:** 10.1016/j.ijscr.2019.04.006

**Published:** 2019-04-06

**Authors:** Mohamad Moussa, Mohamed Abou Chakra

**Affiliations:** a Head of Urology Department, Zahra University Hospital, Beirut, Lebanon; b Department of Urology, Lebanese University, Beirut, Lebanon

**Keywords:** Leiomyosarcoma, Spermatic cord, Scrotum, Paratesticular, Case report

## Abstract

**Background:**

Leiomyosarcoma of the spermatic cord is a rare entity. It presents as a firm and painless intrascrotal mass. For diagnosis radiological methods such as ultrasound, CT or MRI are necessary. Radical orchiectomy is the gold standard of treatment.

**Case description:**

We reported a one case of a 66-year-old man that he has suffered from a painless hard mass in the right hemiscrotum since three years. Ultrasound revealed a paratesticular mass. Further workup revealed no distant metastasis before surgery. Right radical orchiectomy was performed, the final pathologic examination showed a leiomyosarcoma of the spermatic cord. The patient remained disease-free twelve-month during follow up after surgery.

**Discussion:**

Leiomyosarcoma of the spermatic cord is a rare condition, it arises from mesenchymal cells of the spermatic cord and occurs mainly in elderly patients. The diagnosis of spermatic cord leiomyosarcoma is difficult if based only on imaging procedures, it is revealed by histological examination post surgery. No treatment protocol has yet been established for paratesticular leiomyosarcoma due to the rarity of the disease. Standard treatment is radical orchidectomy with high ligation of the spermatic cord. The benefit of adjuvant chemotherapy, radiotherapy, and retroperitoneal lymphadenectomy are not well understood and may differ according to the surgeon's preferences.

**Conclusion:**

The lack of such cases results in the challenging nature of the diagnosis and treatment of leiomyosarcoma of the spermatic cord. Additional studies are needed to better define optimal management strategies.

## Highlights

• Leiomyosarcoma of the spermatic cord is a rare entity.
• The diagnosis of spermatic cord leiomyosarcoma is difficult based on imagery alone.
• The standard treatment is radical orchidectomy with high ligation of the spermatic cord.

## 1 Introduction

Primary paratesticular tumors are rare, accounting for 7–10% of all intrascrotal tumors. They are commonly grouped according to their location: testicular tunica, epididymis, or spermatic cord. Primary spermatic cord tumor is the most common tumor of the paratesticular region. Soft-tissue sarcomas of the genitourinary tract are extremely rare malignancies, the most common reported malignant histological types are liposarcomas, leiomyosarcomas, rhabdomyosarcomas, and fibrosarcomas. Leiomyosarcoma of the spermatic cord is infrequent, about 110 cases have been reported in the literature [1]. Preoperative diagnosis of paratesticular leiomyosarcoma is challenging and usually presented with firm-painless intrascrotal mass. Diagnostic tests include sonography Ultrasound, CT, or MR. Treatment recommendations are based on case reports, small case series and should be studied further.

This work has been reported in accordance with the SCARE criteria [2].

## 2 Case report

A 66 years old male patient, known to have hypertension showed up with a painless lump in the right hemiscrotum that he has been suffering from since 3 years. He had no history of testicular trauma or infection or lower urinary tract symptoms. Examination showed a well-delimited mass of 5 cm, firm, non-mobile with irregular border over the right spermatic cord. All blood examinations were normal. The serum levels of alpha-fetoprotein, beta-hCG, lactate dehydrogenase were within the normal limits. The patient denied scrotal pain, hematuria, dysuria, fever or chills.

**Fig. 1:**
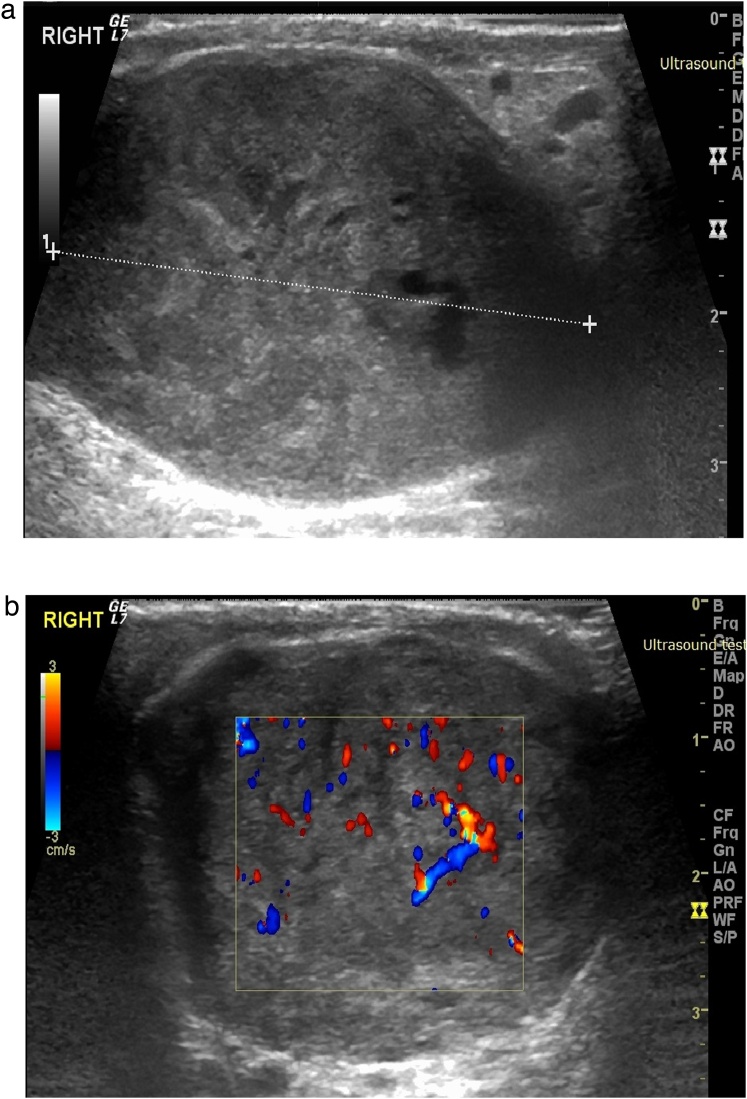
A: Scrotal ultrasound showing a well-circumscribed mass of mixed heterogeneous echogenicity on the right side. B: Doppler ultrasound demonstrating increased vascularity of the lesion.

Ultrasound of the scrotum (Fig. 1-A) showed 4 × 3 × 4 cm, circumscribed heterogeneous solid extra-testicular mass located above the right testicle with increasing vascularity (Fig. 1-B) suggesting a neoplastic mass. The epididymis seems to be preserved, the right testicle shows homogenous echo structure. CT-scan of chest, abdomen, and pelvis were negative for local or distant metastasis.

**Fig. 2:**
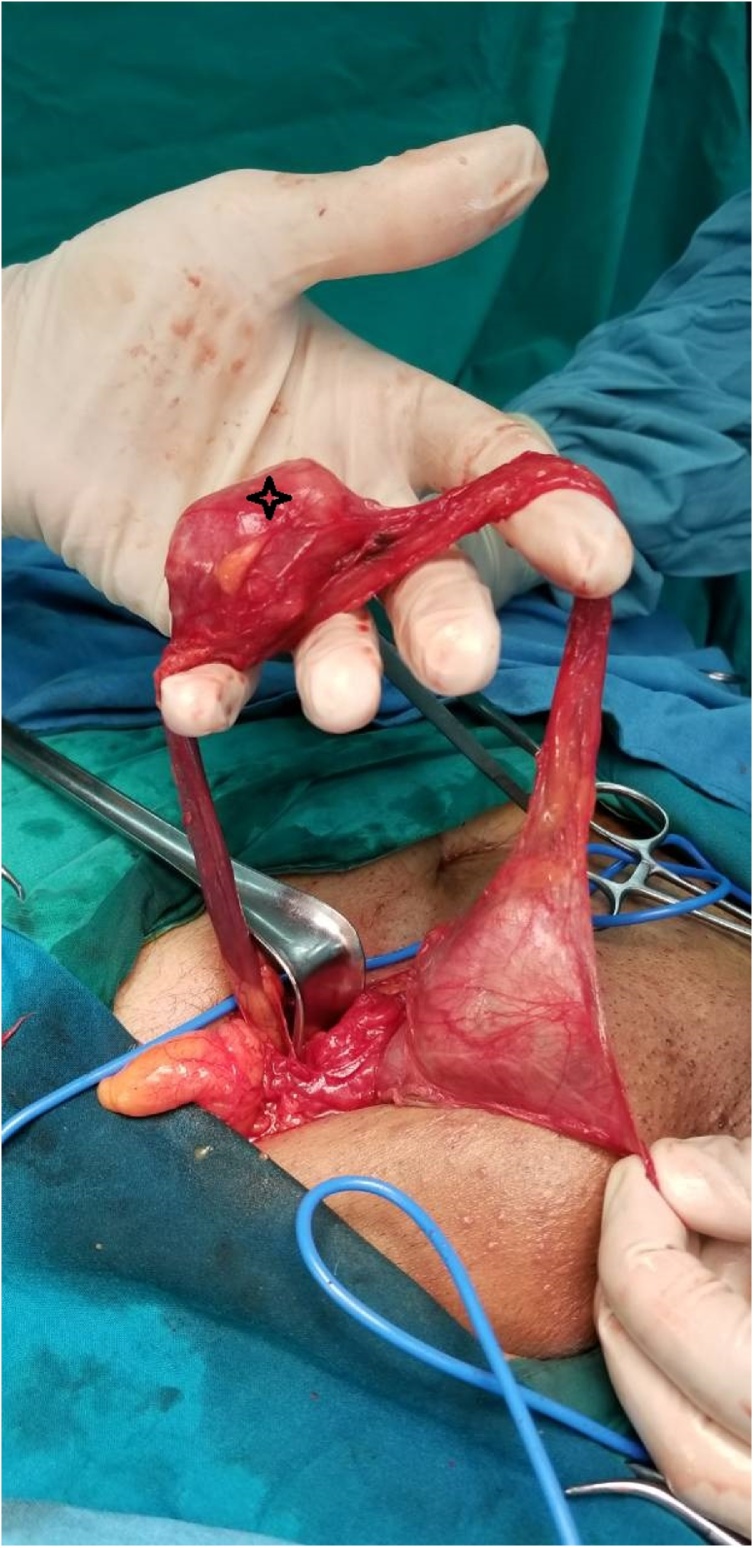
Intraoperatively: asterisk: Spermatic cord mass.

**Fig. 3:**
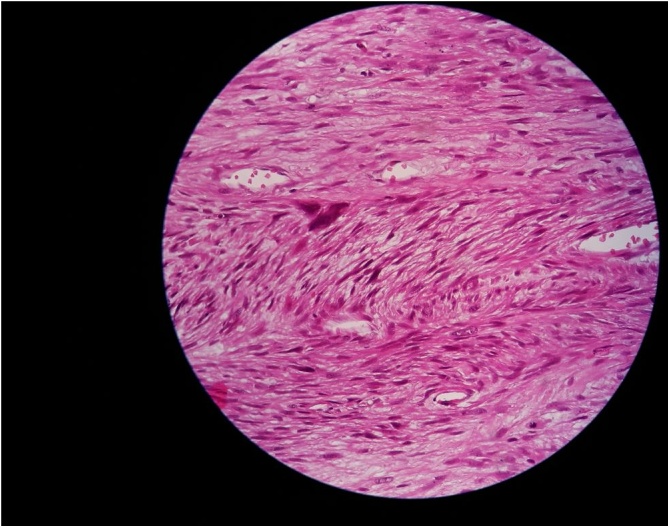
Interlacing fascicles of spindle cells with high cellularity and atypical mitosis.

Right radical orchidectomy along with excision of the spermatic cord mass was performed. During the surgery a mass is seen arising from the upper part of the cord while the testis and epididymis were separate from the lesion (Fig. 2). Microscopic examination (Fig. 3) of the well -delineated mass demonstrated fascicles of spindle cells with eosinophilic cytoplasm of probable smooth muscle origin. Focal areas with pleomorphic morphology and hypercellularity with two to three mitoses per high power field (2-3/HPF) are present. The immunohistochemistry tested positive for Vimentin and Actin. Definitive pathological diagnosis of this patient revealed a leiomyosarcoma of the right spermatic cord with negative surgical margins. The clinical and radiologic follow-up with thoraco-abdominopelvic CT for 6 and 12 months shows no signs of local recurrence and distant metastases.

## 3 Discussion

Leiomyosarcoma of the spermatic cord is a rare entity, only about 110 cases have been reported in the literature. A review of 10 series of paratesticular sarcomas in adults showed that leiomyosarcoma is the most commonly reported histological variety, with a peak incidence in the sixth and seventh decade [1]. The leiomyosarcoma of the spermatic cord mainly derives from the undifferentiated mesenchymal cells of cremaster muscle and ejaculatory duct.

Leiomyosarcoma is subdivided into different groups on the basis of degree and distribution of organ or tissue involvement. The three main subcategories are leiomyosarcoma of deep soft tissue, cutaneous/ subcutaneous tissue and vascular origin. The American Joint Committee on Cancer (AJCC) classifies spermatic cord leiomyosarcoma as deep tissue. The grading of paratesticular leiomyosarcoma is based on the evaluation of the number of mitoses, the percentage of necrosis and severity of nuclear pleomorphism, those factors are important in the biologic behavior of the tumor [3]. These tumors have an extremely variable prognosis with a wide spectrum of behavior. Painless intra-scrotal paratesticular firm mass is the most common presentation of leiomyosarcoma of the spermatic cord.

Ultrasonography (US) is the primary modality for imaging scrotal lesions. Intratesticular versus extratesticular pathologic conditions can be differentiated with 98%–100% sensitivity. Sarcomas of the spermatic cord have also been reported to be hyperechoic and therefore echogenic masses cannot be dismissed, MR imaging may be very helpful in those tumors [4].

The definitive diagnosis is given by histology. Immunohistochemical demonstration of muscle-specific actin (HHF-35), α-smooth muscle actin, desmin, and h-caldesmon indicates smooth muscle differentiation. Several histological parameters are used to evaluate malignant potential in smooth muscle tumors such as the tumor size, cellular atypia, hypercellularity, mitotic index, atypical mitosis, coagulative necrosis, location of the tumor, tumor invasion, and metastasis. The most reliable indication of malignant potential is the mitotic index (mitotic figures per 10 HPF) (MI), which MI > 10 suggests a malignancy [5].

No treatment protocol has yet been established for paratesticular leiomyosarcoma due to the rarity of the disease. The standard treatment is radical orchidectomy with high ligation of the spermatic cord. Aggressive surgical strategies are therefore recommended in the treatment of those tumors. A decrease in local recurrence has been observed in patients who underwent re-operative wide excision after a prior incomplete resection [6].

The role of prophylactic lymph node dissection remains unclear. There is insufficient evidence at present to suggest that prophylactic para-aortic and pelvic nodal dissection prevents relapse or improves the prognosis of patients with spermatic cord leiomyosarcoma [7].

The role of radiotherapy remains controversial. There is increasing consensus that leiomyosarcoma of all grades and histology should receive adjuvant radiotherapy [8]. A study from Massachusetts involved 18 patients with spermatic cord sarcoma who were divided into 2 groups of 9 patients. One group had surgery and the other group had surgery plus radiation. Of the 9 patients treated with radical orchidectomy alone, 5 developed loco-regional failure, 2 of which were limited to lymph nodes. In contrast, there were no loco-regional recurrences among the 9 patients who received adjuvant radiation to the regional lymph nodes [9].

Conventional adjuvant systemic chemotherapy has no exact efficacy for spermatic cord leiomyosarcoma. At present, the role of chemotherapy remains controversial and restricted to the presence of metastatic disease [10].

In this particular case, we learned that leiomyosarcoma should be considered one of the differential diagnoses for a hard lump in the cord and treatment could be only by radical orchiectomy without adjuvant therapy. The patient may have a good response and disease free-survival.

## 4 Conclusion

The diagnosis and management of leiomyosarcoma of spermatic cord remain challenging with no clear data due to the rarity of the disease. This condition should be included in the differential diagnosis of elderly males presenting for a scrotal mass. Radical orchiectomy remains the gold standard for the management of patients with leiomyosarcoma of the spermatic cord. More research to develop evidence is needed to determine the best treatment for this disease.

## Conflicts of interest

None identified.

## Funding

No funding.

## Ethical approval

Ethical approval is not required by our institution.

## Consent

Written informed consent was obtained from the patient for publication of this case report and accompanying images.

## Author contribution

Mohamed Abou Chakra, Mohamad Moussa: case report design.

Mohamad Moussa and Mohamed Abou Chakra: contributed to the manuscript preparation.

Mohamed Abou Chakra, Mohamad Moussa: followed up the patient and revised the manuscript.

Mohamed Abou Chakra, Mohamad Moussa: approved the final manuscript.

## Registration of research studies

Not applicable, case report.

## Guarantor

Mohamed Abou chakra.

## Provenance and peer review

Not commissioned, externally peer-reviewed.
